# Supplementary material for: Decreased Serum Stromal Cell-Derived Factor-1 in Patients with Familial Hypercholesterolemia and Its Strong Correlation with Lipoprotein Subfractions
Source: Int J Mol Sci. 2023 Oct 18;24(20):15308. doi: 10.3390/ijms242015308 (PMC10607113; doi:10.3390/ijms242015308)
Supplement: Supplementary file 1 [file ijms-24-15308-s001.zip › ijms-2620629-supplementary.pdf]

Supplementary Table S1. Results of univariate analysis between clinical variables and serum stromal derived factor-1 (SDF-1) in patients with heterozygous familial hypercholesterolemic (HeFH) and controls.

	**HeFH**		**Controls**	
	**r**	**p-value**	**r**	**p-value**
Age (yrs)	0.11	0.374	-0.02	0.930
Gender (m/f)	0.22	0.068	0.30	0.143
Body mass index (kg/m^2)	-0.10	0.488	-0.18	0.379
Fasting glucose (mmol/L)	-0.05	0.691	0.06	0.733
Cholesterol (mmol/L)	0.03	0.783	-0.21	0.255
lg Triglyceride (mmol/L)	-0.22	0.069	**-0.36**	**0.044**
LDL-C (mmol/L)	0.08	0.535	-0.30	0.102
ApoB100 (g/L)	-0.16	0.188	-0.37	0.074
VLDL (mmol/L)	-0.17	0.189	**-0.44**	**0.014**
IDL (mmol/L)	0.11	0.383	0.01	0.967
Large LDL (mmol/L)	0.19	0.131	-0.35	0.057
Mean LDL size (mmol/L)	**0.26**	**0.041**	0.33	0.069
HDL-C (mmol/L)	0.12	0.320	0.18	0.339
ApoA1	0.06	0.626	0.04	0.871
Large HDL (mmol/L)	**0.27**	**0.032**	0.19	0.297
Intermediate HDL (mmol/L)	0.23	0.067	0.23	0.216
Small HDL (mmol/L)	-0.14	0.278	-0.17	0.375
Oxidized LDL (U/L)	-0.02	0.856	-0.29	0.113
lg MPO	-0.10	0.395	0.01	0.960

Significant correlations ($p<0.05$) are marked in bold print. Abbreviations: ApoA1: apolipoprotein A1; ApoB100: apolipoprotein B100; HDL-C: high-density lipoprotein cholesterol; IDL; intermediate–density lipoprotein; LDL-C: low-density lipoprotein cholesterol; MPO: myeloperoxidase; oxLDL: oxidized LDL; VLDL; very-low density lipoprotein.

Notes: The relationship between variables and SDF-1 was analyzed with Pearson's univariate test.
